# A Highly Efficient Adsorbent Cu-Perusian Blue@Nanodiamond for Cesium in Diluted Artificial Seawater and Soil-Treated Wastewater

**DOI:** 10.1038/s41598-018-24129-0

**Published:** 2018-04-11

**Authors:** Kazuko Matsumoto, Hideyuki Yamato, Seishiro Kakimoto, Takeshi Yamashita, Ryutaro Wada, Yoshiaki Tanaka, Masakazu Akita, Tadamasa Fujimura

**Affiliations:** 1 Department of Applied Chemistry, Tokyo University of Technology, 1404-1, Katakura-cho, Hachioji, Tokyo, 192-0982 Japan; 2 Vision Development Co. Ltd., 2-8-21, Kikuya bld., Kyobashi, Chuo-ku, Tokyo, 104-0031 Japan; 3 Mechanical Engineering Research Laboratory, Kobe Steel, Ltd., 1-5-5, Takatsukadai, Nishi-ku, Kobe, 651-2271 Japan; 4 Natural Resources & Engineering Business, Kobe Steel, Ltd., 9-12, Kita-Shinagawa, 5-Chome, Shinagawa-ku, Tokyo, 141-8688 Japan; 5 Nuclear & CWD Division, Natural Resources & Engineering Business, Kobe Steel, Ltd., 2-7, Iwaya-Nakamachi, Nada-ku, Kobe, 657-0845 Japan

## Abstract

A new adsorbent Cu-Perussian blue@Nanodiamond (Cu-PB@DND) for Cs^+^ removal was prepared and characterized with IR, SEM, X-ray diffraction, particle size analysis, and zeta-potential. The adsorbent consists of a core of aggregated detonation nanodiamond (DND) particles with the surface treated with Cu-PB. Cesium adsorption was studied in two modes; a co-precipitation mode and a batch mode. In the co-precipitation mode, DND, CuCl_2_, and K_4_[Fe(CN)_6_] were added sequentially to a Cs^+^ solution in diluted artificial seawater. In the batch mode, adsorbent Cu-PB@DND was dispersed into a Cs^+^ solution with stirring. The distribution coefficient (K_d_) of the co-precipitation mode was 8.8 × 10^7^ (mL/g) at Cs^+^ 6.6 ppm in 0.07% seawater. The K_d_ value of the batch mode was 1.3 × 10^6^ (mL/g). Precipitation of Cs^+^-incorporated particles was complete, and post filtration was not necessary. Excess copper and iron ions were completely removed and were not detected in the supernatant. The adsorption data for Cu-PB@DND were analyzed by assuming Langmuir isotherm and a good fit was obtained with a maximum adsorption capacity Q_max_ of 759 mg/g. The co-precipitation method was also applied to soil-treated wastewater.

## Introduction

The tragic disaster of the earthquake and Tsunami in Fukushima Daiichi nuclear power plant in 2011 urged us to develop a new efficient adsorbent for radioactive elements. Among them, ^137^Cs is in particular a threat to environment, since it emits hazardous β-particles and strong γ-ray with a long half-life (30.4 years). In addition, Cs^+^ has high transportability via the atmosphere, high solubility in aqueous media, and strong and persistent adsorption to soil^1^. In the accident, seawater was used to cool down the out-of-control power plant, which made removal of Cs^+^ very difficult. A new adsorbent with high selectivity and high adsorption capacity to Cs^+^ is desired. During decontamination process and subsequent storage of used adsorbents, deterioration of the adsorbent by radioactivity is also a serious problem. A new adsorbent must have high resistance to radioactivity for safe long-time storage.

Detonation nanodiamond (DND) is a new nanocarbon material, synthesised from a mixture of explosives and carbon materials such as graphite and active carbon. With momentary high pressure by detonation in a closed steel chamber, black soot is collected. The soot contains aggregated spherical primary particles of nanodiamond with diameter of 4–6 nm, which is imbedded in a large amount of amorphous carbon and graphite. After strong oxidative purification of the soot, most non-diamond components are removed to give DND particles of 200 nm or less in diameter. This spherical particles are still aggregate of several to many primary nanodiamond particles, and each particle is solidly covered with graphite-like carbon thin layers. Such aggregated DND can be dispersed in water, due to covalently-bound surface polar functional groups, such as carboxyl, hydroxyl, and ketonyl groups^2–8^. In addition, the aggregated DND contains substantial amount of water (10–30 wt%) between the graphite layers or in the interface of surface graphite and nanodiamond core. The water is retained very tightly in the particles by adsorption or some other chemical interactions, and high temperature and vacuum are necessary to remove the water. High specific surface area of DND with surface polar functional groups, high dispersion in water, easy precipitation in the presence of metal-ions, and the rugged and porous surface structure of DND particles suggest that DND might be an effective adsorbent for Cs^+^ in seawater and dirty wastewaters.

So far only a few applications of metal ion adsorption by DND are reported in literatures. One is on Cs^+^ adsorption, and the adsorption capacity was dependent on the surface treatment of DND. The adsorption capacity of 0.7 mmol Cs^+^/g is reported^9^. Selective adsorption of tungstate anion by DND is reported as a tool for pre-concentration of tungstate in ICP-AES (Inductively Coupled Plasma-Atomic Emission Spectroscopy) measurement^10^. In this application, DND did not adsorb metal cations, owing to the positive zeta-potential of the DND, and high selectivity for tungstate was realized. Zeta-potential of DND varies from negative to positive, depending on the DND source and purification and surface treatment^4,6^. Several mechanisms are conceivable for metal ion adsorption; (1) coordination of metal ions onto the surface functional groups, (2) electrostatic attraction and ion-exchange interaction on the functional groups, (3) physical adsorption and intercalation in the surface graphite layers, (4) crosslinking between several DND particles, leading to aggregation and precipitation. However, real metal adsorption occurs through complex mechanisms and no detailed study has been carried out.

Prussian Blue (Fe_4_[Fe(CN)_6_]_3_ or K_x_Fe_y_[Fe(CN)_6_]) (PB) is a well-known adsorbent highly efficient and selective to Cs^+ ^^11–13^. The strong adsorption and high selectivity arises from the face-centered cubic lattice consisting of -Fe-CN-Fe-NC-Fe- infinite chains. The K^+^ ions in the cubic voids of the lattice are exchangeable with Cs^+^, and once Cs^+^ enters the void, the cation is stably trapped, because the hydrated ionic radius of Cs^+^ fits tightly to the size of the void. Compared to other Cs^+^ adsorbents, such as zeolite and other inorganic adsrobents^14^, biomass^15^, and clay minerals^16^, PB has high capability and selectivity to Cs^+^. The adsorption capability of PB for alkali metal ions is in the order of Cs^+^ ≫ K^+^  ≧ Na^+^. In addition to original PB, Cs^+^ adsorption is reported also to PB analogues containing various hetero metal ions such as Ni^2+^, Cu^2+^, Co^3+^, Mn^2+^ and Zn^2+ ^^17–32^ as partial substituents for Fe ions in the lattice. It is not easy to decide which hetero metal ion is better than others for Cs^+^ adsorption, since literature experiments were carried out under different conditions and on various sample matrices.

Although PB and its analogues have excellent adsorption capability, PB prepared in solution is usually very fine powder, and complete precipitation after Cs^+^ adsorption is very difficult. For efficient Cs^+^ removal, fine Cs^+^-adsorbed PB powder must be removed from the solution. Milli- or nano-pore filter is usually necessary for such purpose. However, filtration requires long-time labor, and attempts have been made to transform PB particles into larger particles or beads, i.e., PB was transformed into crystals in hydrogel^18^ or was attached to surface amino groups^31^ or macrocyclic ligands^33^ on silica beads, or PB was mixed with polyvinyl alcohol to make beads^21^. Silicate beads containing PB or its analogues are also reported^26^. Mushroom biomass is also reported as a support for PB^19^.

In the present study, DND is used as a support for a PB analogue, Cu-Perussian blue, Cu-PB. Surface of DND was modified with Cu-PB, and its Cs^+^ removal performance was evaluated in diluted artificial seawater and soil-treated wastewater. The surface modified DND, Cu-PB@DND, was characterized by IR, SEM, particle-size distribution analysis, and zeta-potential.

Several nanocarbon materials have been reported to adsorb Cs^+^ in their unmodified or PB-modified forms. Graphene and graphene oxide exhibit excellent adsorption capability for metal ions such as Cu^2+ ^^34^, Co^2+ ^^35^, and Cd^2+ ^^35^, owing to the high specific surface area (~2000 m^2^/g), compared to other nanocarbon materials (CNT (carbon nanotube) 120–500 m^2^/g and DND 180–320 m^2^/g). For Cs^+^ removal, only moderate adsorption capability is reported for graphene oxide^36^, however, the adsorption capability is significantly enhanced by surface modification with PB^37^. Both plain CNT and PB-modified CNT are effective for Cs^+^ removal, and PB modification enhances the adsorption property of plain CNT^38^. However, distribution coefficient (K_d_) of the PB-modified CNT is still moderate (1300 mL/g), compared to other PB-modified non-nanocarbon adsorbents. Since DND is dispersive in water and tends to aggregate and precipitate in the presence of metal ions, DND seems a promising adsorbent for Cs^+^. The high chemical and physical stability of carbon materials in general in radioactive environment also tempted us to study Cs^+^ adsorption performance of DND and PB-modified DND. In literatures, SiO_2_ nanoparticle is reported as PB support, but other inorganic particles such as TiO_2_ is not reported, presumably because of the photochemical reactivity and instability. Nanocarbon materials are highly preferable as PB supports because of their high chemical stability and radiation stability.

## Results and Discussion

### Preparation and property of Cu-PB@DND

**Figure 1:**
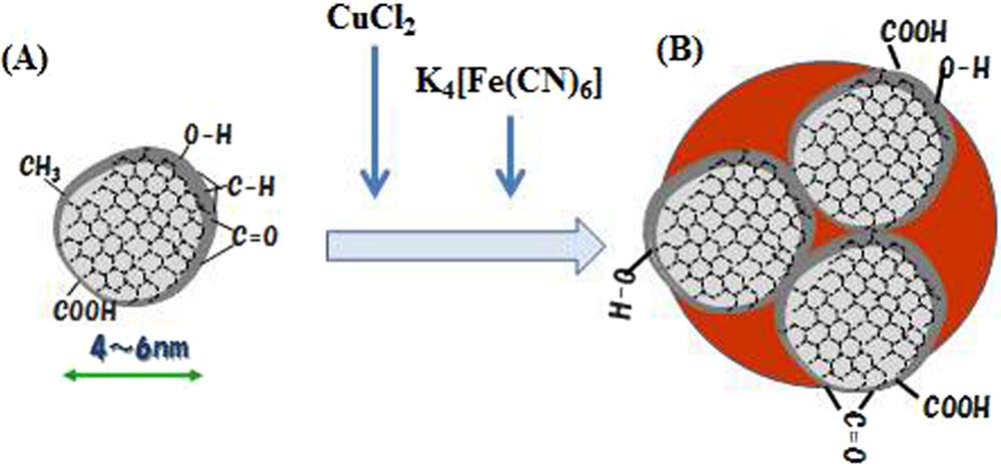
A schematic drawing of the preparation and structure of Cu-PB@DND. (**A**) DND, (**B**) Cu-PB@DND containing aggregated DND particles with surface modification of red-brown Cu-PB.

Schematic drawings of the structure of a DND particle and preparation of Cu-PB@DND are shown in Fig. 1.

For preparation of Cu-PB@DND, CuCl_2_ solution was added to a dispersed solution of DND(II) (see the next IR section for the definition of DND(II)). At this stage, the added Cu^2+^ was adsorbed on DND surface. In the next step, K_4_[Fe(CN)_6_] solution was added to the solution. After a while, red-brown Cu-PB@DND appeared and precipitated in the solution. This adsorbent of Cu-PB@DND at this stage was fine powder, but as shown later, had larger average diameter than the original unmodified DND(II). The supernatant was removed by pipetting and the Cu-PB@DND residue was used in wet state for Cs^+^ adsorption experiment. For spectroscopic characterization, the adsorbent was measured after drying.

### IR spectrum, zeta potential, SEM, particle size, and elemental analysis of DND and Cu-PB@DND

**Figure 2:**
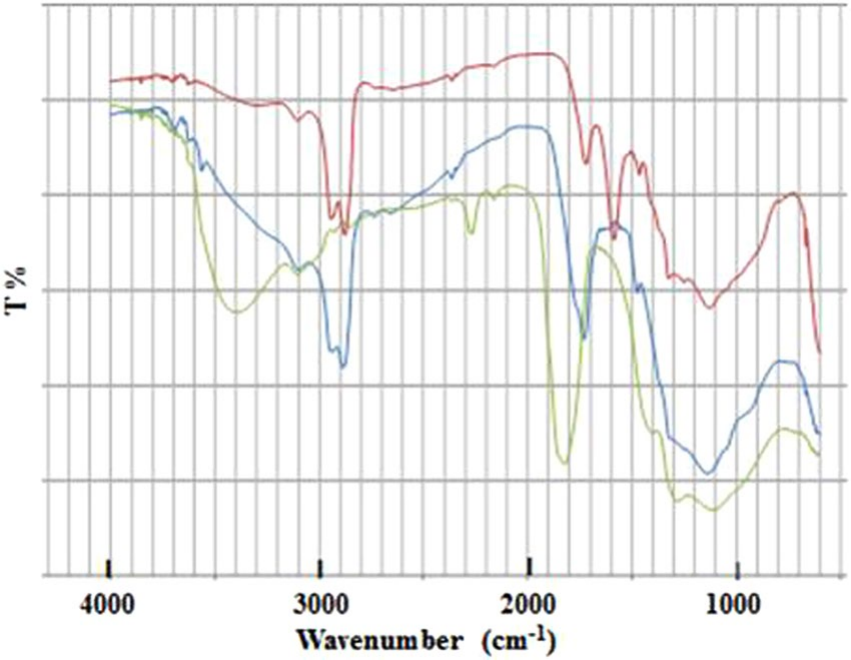
IR spectra of DNDs. blue line: original DND(I), green line: DND(II), i.e., air- heated DND(I), red line, DND(III), i.e., NaOH-treated DND(I).

**Figure 3:**
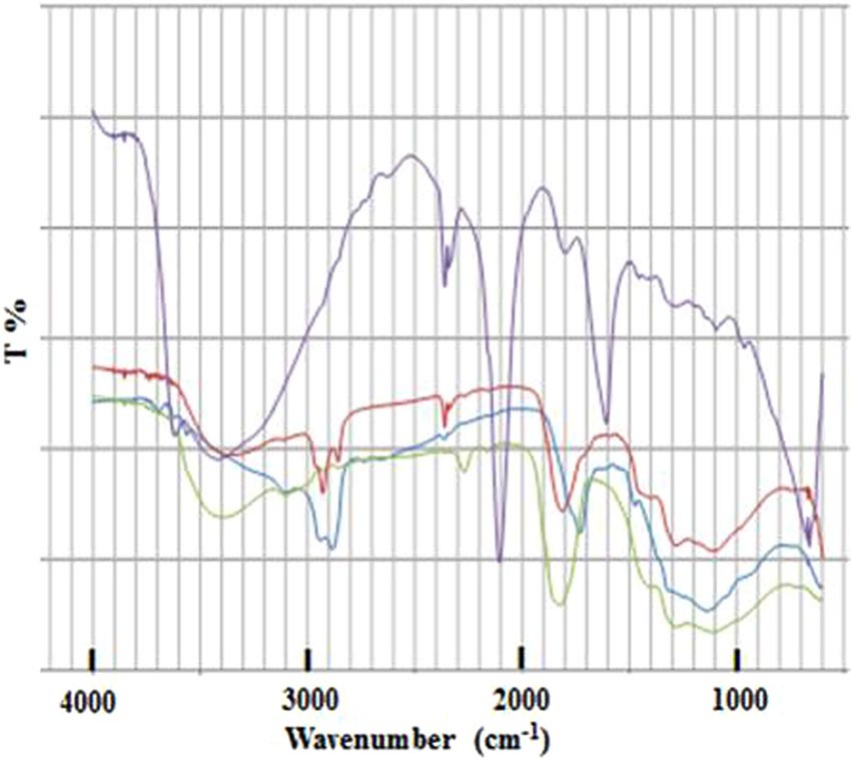
IR spectra of DNDs. blue line: DND(I) original, green line: DND(II) prepared by heating in air in 2011, red line: DND(II) prepared by heating in air in 2016, purple line: Cu-PB@DND.

The IR spectra of DND(I) (original DND), DND(II) (air-heated DND(I)) and DND(III) (NaOH-treated DND(I)) are shown in Fig. 2. The IR spectra of DND(I), DND(II) air-treated in 2011, DND(II) air-treated in 2016 and Cu-PB@DND are shown in Fig. 3. All of the three spectra in Fig. 2 have common absorption bands at 3200–3600 cm^−1^, 2900 cm^−1^, 1700–1800 cm^−1^, and 1600 cm^−1^, but the relative intensities of the bands vary depending on the surface treatment. Such treatment-dependent IR property is already known for DNDs with different surface treatments^6,39–41^. Referenced to the previous assignment in the literatures, the bands were assigned as follows: 3400 cm^−1^ is O-H stretching of water and phenols, 2900 cm^−1^ is C-H stretching, 1700–1800 cm^−1^ is C=O of ketones and carboxylic groups, and 1600 cm^−1^ is several C-O groups such as esters and lactones or water molecules in outer graphite-like layers. Surface water amount usually varies significantly depending on the drying condition and chemical surface treatment. Water molecules in DND are remarkably strongly associated with DND, and are probably bound between the outer graphite-like layers or are associated with the interface of nanodiamond core and outer graphite-layers. Extensive depletion of the water needs vacuum and elevated temperature^42^. Therefore, IR spectra were measured at 120 °C in N_2_ in the present experiment. It is obvious in Fig. 2 that sintering of DND(I) in air increases water amount probably bound to surface graphite layer, whereas NaOH treatment decreases water amount and most surface functional groups except C-H are lost. The surface of DND(III) has less functional groups and is thus more hydrophobic than DND(I) and DND(II), which suggests that DND(III) would be less favorable for metal ion adsorption than others.

Among the two air-treated DNDs in Fig. 3, significant difference is observed at the band 1900 cm^−1^. The band still remains in DND(II) treated in 2016, but is almost lost by air-heating in 2011. The reason for such a significant difference is not known. The IR spectrum of Cu-PB@DND shows new bands, in addition to those of original DND(II), at 2100 cm^−1^, 1600 cm^−1^, and 670 cm^−1^. These three bands are also reported for Cu^II^_3_[Fe^III^(CN)_6_]_2_ · xH_2_O,^30^ Cu^II^_2_[Fe^II^(CN)_6_] · xH_2_O,^30^ and PB on graphene^37^, and are assigned to C≡N stretching (2100 cm^−1^) and Fe-CN-Fe bending (670 cm^−1^). The band at 1600 cm^−1^ has not been clearly assigned in literatures, but is always observed weakly or moderately in PB and Cu-PB.

Zeta potentials of DNDs in water were + 46 mV for DND(I), −14 mV for DND(II) and + 4 mV for DND(III).

**Figure 4:**
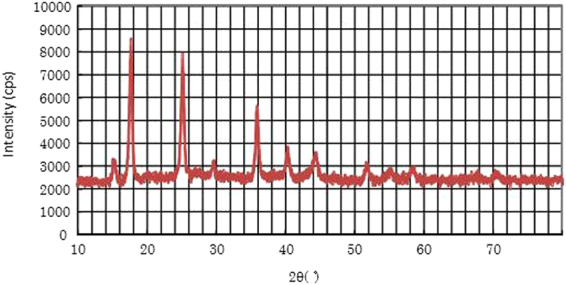
X-ray diffraction pattern of Cu-PB@DND.

The X-ray diffraction pattern of Cu-PB@DND is shown in Fig. 4. All of the peaks correspond to K_x_Cu_y_[Fe(CN)_6_] · xH_2_O or PB reported in literatures^30,38,43–46^. Clear peak assignment is not reported, since relative peak intensities and positions vary depending on the amount of crystal water and hetero metal ions^30,37,38^. The two compounds give almost an identical X-ray diffraction pattern and are not distinguished in the literatures. Peaks of DND(II) are not observed in Fig. 4. Non-PB-modified DNDs usually give diamond peaks at 43 and 72 degree and a small graphite peak at 27 degree^4,6^. In Fig. 4, these DND peaks are not observed, maybe because only the thin surface layer of Cu-PB in Cu-PB@DND contributes to the X-ray diffracrion pattern. Similarly, only PB X-ray diffraction peaks are observed in other PB modified CNT, graphene and graphene oxide, for Cs^+^ adsorption^28,38,44^. Existence of DND in Cu-PB@DND has been proved in the IR spectrum in Fig. 3, since longer wavelength of IR can penetrate into deeper sites of particles, and thus give spectrum peaks of both surface Cu-PB and core DND.

**Figure 5:**
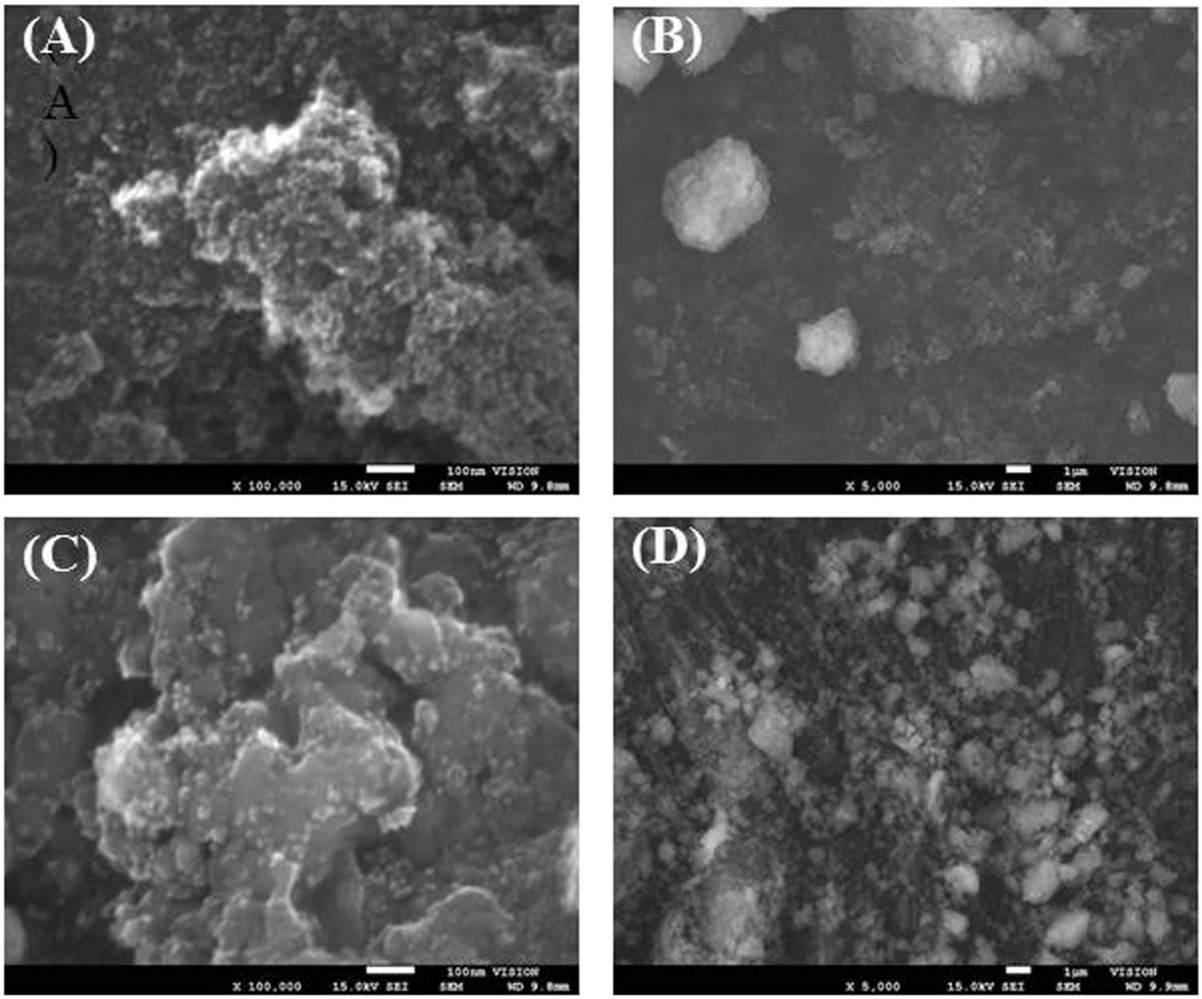
The SEM images of (**A**) DND(II) with the bar 100 nm, (**B**) DND(II) with the bar 1 μm, (**C**) Cu-PB@DND with the bar 100 nm, (**D**) Cu-PB@DND with the bar 1μm.

The SEM images of DND(II) and Cu-PB@DND are shown in Fig. 5. In Fig. 5(A and B), many aggregated spheres are observed, onto which smaller particles and primary particles of DND less than 10 nm are attached. In Fig. 5(B), the approximate aggregate size ranges from less than 100 nm to more than several μm. Since primary particles of DND in literatures are about 4–6 nm in diameter, and the particle size distribution in the initial dispersion of DND(II) is about 100 nm to 1 μm (Fig. S1), the aggregation in Fig. 5(A and B) would have been caused by drying. The EDS (energy dispersive semiconductor) analysis of DND(II) and Cu-PB@DND shows the elemental composition (%) of DND(II) as C 90.7, O 7.9, Zn 0.4, Fe 0.4, Si 0.3, Al 0.2, S 0.2 and Cu 0.1, and that of Cu-PB@DMD as C 54.9, Cu 21.7, Fe 13.2, N 8.3, O 1.9, and K 0.2. The elemental values of the same sample measured with ICP-MS using a nebuliser for dispersion solution, after HNO_3_ digestion for 2 hrs, were less than 1/5 of the EDS values. This is due to the fact that for EDS, part of the water in DND is lost by vacuum, and for ICP-MS, most of the nanodiamond particles remain undecomposed in solution even after strong acid digestion, and is only partially introduced into the plasma with a conventional nebulizer, because of the high specific density of diamond particle (ca. 3.5) with heavy metal impurity. Many literatures report impurity values obtained with ICP-MS, but these must be considered with care.

The SEM images in Fig. 5(C and D) show that the average particle size is significantly increased by Cu-PB modification. The average particle size of the adsorbent dispersed in water was measured with LSD (laser scattering dynamics) (Fig. S1). Before PB modification, the average particle diameter of DND(II) was 0.2 ± 0.4 μm, whereas it is 5 ± 4μm after Cu-PB modification.

### Comparison of DND(I), DND(II) and DND(III) in Cs^+^ Removal

Removal efficiency, R_eff_ (%) (R_eff_ = 100 × (C_0_ –C_e_)/C_0_, where C_0_ and C_e_ are original and equilibrium Cs^+^ concentration in solution, respectively), was compared for DND(I), DND(II) and DND(III), by using a 10 ppm Cs^+^ solution in diluted artificial seawater (0.07%). The Cs^+^ adsorption and precipitation experiment was carried out by addition of DND to a Cs^+^ solution to 0.25 μg/L. After the solution was stirred for 2 hours and left standing overnight, Cs^+^ concentration in the supernatant was measured with ICP-AES. The removal efficiencies were as follows: unmodified DND(I) 22%, air-heated DND(II) 38%, and NaOH-treated DND(III) 13%. Therefore, subsequent experiments were carried out with DND(II). As expected, DND(III) is least effective, since it has the fewest number of polar functional groups, as observed in the IR spectrum (Fig. 2). Air-heated DND(II) having more water molecules around the diamond core, as observed in Fig. 2, has negative zeta-potential, and thus has the highest removal efficiency.

### Removal of Cs^+^ by Cu-PB@DND

Cesium removal experiment was carried out in two modes: (i) co-precipitation by sequential addition of DND, CuCl_2_ and potassium ferrocyanide. The latter two reagents were added to prepare PB *in situ* in the Cs^+^ solution. (ii) batch treatment of Cu-PB@DND adsorbent in a Cs^+^ solution. The results were evaluated in terms of removal efficiency, distribution coefficient of Cs^+^, capacity of adsorbent, and selectivity to Cs^+^. Detonation nanodiamond (DND) has advantage over other common non-carbon adsorbents that DND is resistant to chemicals, high temperatures up to 450 °C under atmospheric pressure in air and radioactivity.

### Removal of Cs^+^ in diluted artificial seawater with co-precipitation mode

Literatures show that not only PB but also several other PB-analogue complexes [M_x_Fe_1−x_(CN)_6_]^y−^, where M is Ni^2+^, Cu^2+^, Zn^2+^, Co^3+^, and Mn^3+ ^^17–32^, are effective for Cs^+^ removal in freshwater and in some cases in seawater. These complexes having hetero metal M^n+^ ion are synthesised by addition of MCl_2 or 3_ to a solution of potassium ferrocyanide. The removal efficiency for Cs^+^ depends on M^n+^, and also preparation method. Different preparation method gives different particle size, which affects the removal efficiency. In the present experiment, MCl_2 or 3_ was first varied in co-precipitation mode, i.e., DND(II), MCl_2 or 3_, and potassium ferrocyanide were added sequentially in this order to seawater (0.07%) containing 20 ppm Cs^+^. After precipitate of Cs^+^-incorporated M-PB settled, Cs^+^ removal efficiencies were calculated from the Cs^+^ concentration in the supernatant. The results are as follows: Cu^2+^ ( > 99%), Fe^3+^ (92%), and Co^3+^ (87%). Therefore, subsequent experiments were carried out with CuCl_2_. The addition order of the three reagents, DND(II), CuCl_2_ and potassium ferrocyanide, critically affected the removal efficiency, and the addition order as described above gave the best removal. Addition of DND(II) after either one or both of the reagents remarkably decreased Cs + removal efficiency. The final Cs^+^ concentrations in the supernatant of various addition combination of the reagents are listed in Table 1, which shows that all of the three reagents are necessary to achieve the highest efficiently (run 3). The effect of pH was examined at pH 4, 6.8 and 11 by addition of either NaOH or HCl after all the procedure was finished. The removal efficiency did not change significantly, and therefore the following experiments were carried out at pH 6.8 (without any pH adjustment). It is notable that when Cu-PB alone is used for Cs^+^ removal, Cu-PB is effective only in neutral pH region^25,31^. In the present study, no pH dependence was observed both in co-precipitation and batch modes. Obviously the property of DND to adsorb Cs^+^ as well as Cu-PB operates very much favorably to Cs^+^ removal.

**Table 1:** Removal efficiency of Cs^+^ (R_eff_) in 0.07% seawater by addition of DND, CuCl_2_, and K_4_[Fe(CN)_6_] in co-precipitation mode.

run#	Addition conc. and order of reagents (from left to right)			conc. in supernatant (ppm)			R_eff_
	DND (mg/L)	CuCl_2_ (μM)	K_4_[Fe(CN)_6_] (μM)	Cs	Cu	Fe	(%)
1	0	0	0	6.6	N.D.^a^	N.D.	0
2	0.25	0	0	4.1	N.D.	0.03	38
3	0.25	50	25	0.003	N.D.	0.01	>99
4	0	50	25	1.5	1.2	0.14	77

^a^Not detected.

In Table 1, Cs^+^ was removed perfectly when all of DND(II), CuCl_2_ and K_4_[Fe(CN)_6_] were added (run 3). In addition, Cu^2+^ and Fe^3+^ were also efficiently removed from the solution in run 3. This is in contrast to run 4, in which DND(II) was not added, and in this case Cu^2+^ was only partially removed. It is also noteworthy that Fe^3+^ was most efficiently removed when all of the three reagents were added (run 3), but was only moderately removed when DND(II) was not added (runs 2 and 3). It is notable that PB and Cu-PB precipitated completely and Cu^2+^ and Fe^3+^ were not detected in the colorless transparent supernatants, as shown in Table 1. Owing to such superb adsorption and precipitation effect of DND(II), it was not necessary to filter the supernatant to remove residual fine powder. This is a great advantage of DND(II) in real application to seawater and wastewater. The concentrations of Na^+^, K^+^, Mg^2+^ and Ca^2+^ ions in the final supernatants are listed in Table S1, which shows that none of these metal ions of seawater were adsorbed on Cu-PB@DND, i.e. the adsorbent is highly selective to Cs^+^. Such high removal efficiency and high selectivity to Cs^+^ as well as the complete removal of Cu^2+^ and Fe^3+^ ions have not been reported for conventional PB and PB-analogue adsorbents, zeolites and other organic and inorganic adsorbents. The new adsorbent Cu-PB@DND has been found practically very useful, since the adsorbent retains its capability even in diluted artificial seawater and in a wide range of pH. This is obviously in contrast to previous adsorbents, whose Cs^+^ removal efficiency decreases in diluted seawater or the Cs^+^ removal efficiency is reported only in pure water^31,38,45,46^. Since high concentration of Na^+^ (>175 ppm) in solution severely disturbed Cs^+^ measurement on ICP-AES, all of the present experiments were performed in 0.07% artificial seawater, however, it is highly likely that the present method similarly works in more concentrated seawater.

All of the previous Cs^+^ removal methods using PB or PB analogues used filtration for the final supernatant to remove Cs^+^- containing fine particles in the solution. In the present system, such filtration is not necessary, since all of the Cs^+^-adsorbed adsorbent precipitates completely, owing to the effect of DND(II). The remarkable difference of Cs^+^ removal efficiency between run 3 and other runs in Table 1 suggests that DND(II) enhances precipitation of the adsorbent by aggregation. In literatures, several bead materials as PB supports have been reported for Cs^+^ removal, such as silicate^26^, polymer with silicate^27^ and polyvinyl alcohol beads^21^, with always filtration after adsorption. It should also be noted that the concentration of potassium ferrocyanide in Table 1 is approximately 1/100 to 1/10 of those of literature methods. Since PB contains toxic CN^−^, the concentration should be preferably as low as possible.

In Table 1, DND(II) itself adsorbs Cs^+^ to some extent (run 2), but in addition to cation, DND(II) seemingly adsorbs also negatively charged PB and Cu-PB. Zeta potentials of DNDs are different depending on the different surface treatments, and the reported values range from negative to positive^3–5^. Zeta potential is only an average of the whole surface of DND. On the surface, a variety of functional groups having negative, neutral and possibly positive charges is actually distributed, since the functional groups have different pK_a_s and other cations might have neutralised the charges of the functional groups. It is no wonder that both cations and anions are seemingly adsorbed on DND, but it is also possible that Cs^+^ adsorption on DND neutralises the surface charge of DND and facilitates subsequent adsorption of anions. Adsorption of metal ions other than Cs^+^ onto DND in general is an interesting research target.

### Mechanism of Cs^+^ adsorption in co-precipitation mode

The mechanism of Cs^+^ adsorption and precipitation by simple Cu-PB is explained in literatures as follows. In Cu-PB, Cu^2+^ partially substitutes Fe^2+^ sites in the 3-dimensional cubic lattice structure consisting of [Fe(CN)_6_]^4−^, and Cs^+^ ion is adsorbed by being trapped into the void of the cubic lattice [Cu_x_Fe_1−x_(CN)_6_]^4−^ to form the complex with average composition of Cs_2_[Cu_x_Fe_1−x_(CN)_6_], which finally precipitates^18,22,26^. The partial substitution of copper ion with Fe^2+^ ion in the lattice tunes the void volume of the cubic lattice to fit to the hydrated Cs^+^ ion, thus enhancing adsorption efficiency and selectivity to Cs^+^.

**Figure 6:**
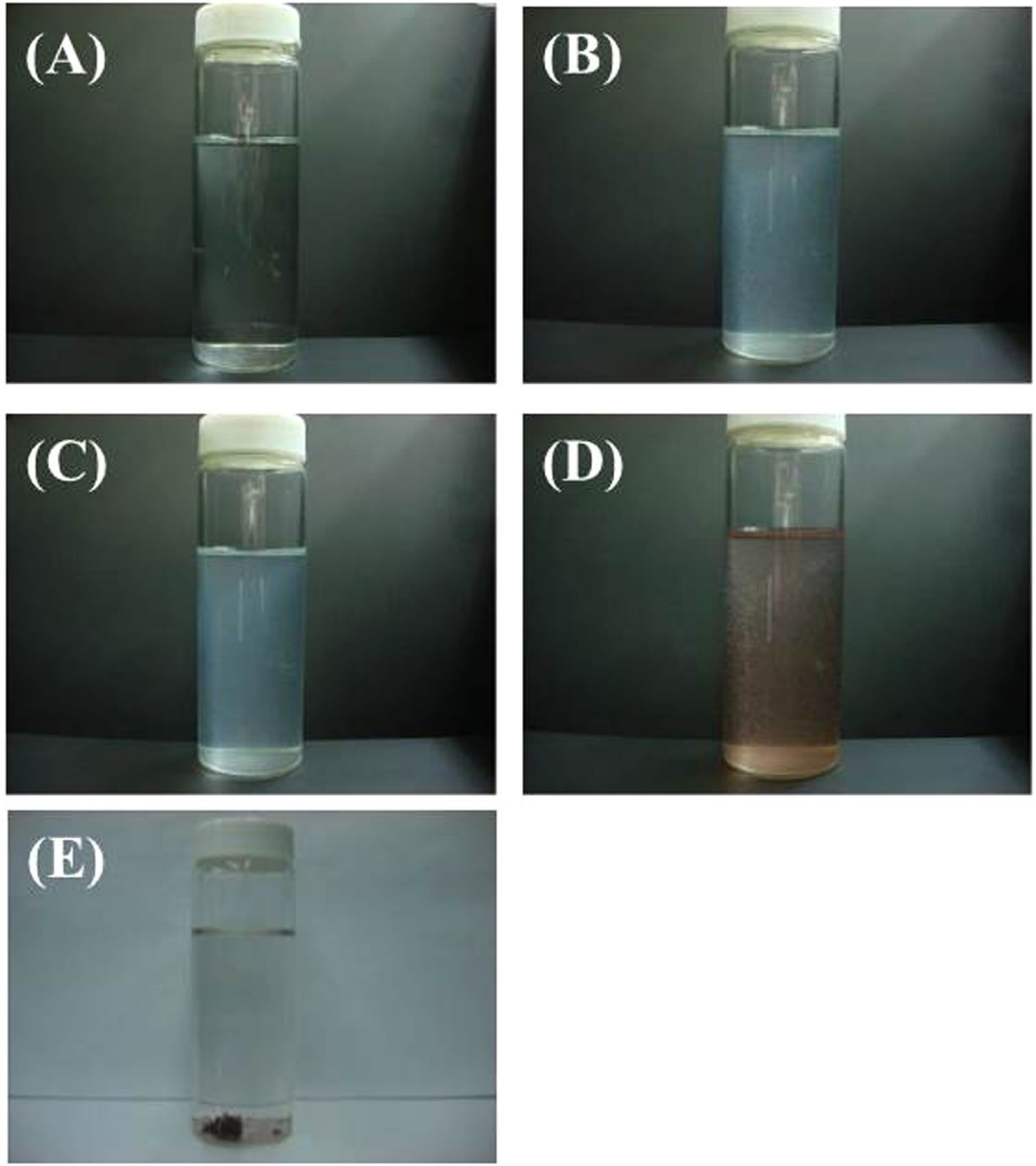
Progress of the co-precipitation reaction of Cs^+^ with DND, CuCl_2_ and K_4_[Fe(CN)_6_]. (**A**) Cs^+^ solution in 0.07% seawater, (**B**) after addition of DND to (**A**), the solution became turbid due to Cs^+^ adsorption and coagulation of DND, (**C**) after addition of CuCl_2_ solution to (**B**,**D**) after addition of K_4_[Fe(CN)_6_] solution to (**C**), the solution turned red-brown, (**E**) after 1 hour, red-brown precipitate settled.

Based on the above reported mechanism for simple Cu-PB, effect of DND in the present co-precipitation method is considered as follows. From run 2, DND itself has adsorption function for Cs^+^, which is probably due to metal cation coordination to surface functional groups of DND, and/or electrostatic attraction between metal cations and DND having negative zeta-potential. In contrast, single digit DND having positive zeta potential of +55 mV is reported to adsorb only negative ions, and is a very selective adsorbent to tungstate anion^10^. For our DND, both positive (Cs^+^ and Cu^2+^) and negative (ferrocyanide) ions seem to be adsorbed and are finally precipitated as Cs^+^-incorporated Cu-PB@DND. The adsorption behavior of DND can be explained that Cu^2+^ adsorbed on DND reacts with ferrocyanide anion added in solution to form Cu-PB on the surface of DND. In such a reaction, electrostatic repulsion between the negative DND and ferrocyanide anion would be mitigated by Cu^2+^ on the DND surface and by the formation of energetically favored Cu-PB. Cesium cation adsorbed on DND would be incorporated into Cu-PB during the formation reaction. In contrast, when independently pre-prepared Cu-PB was added to a Cs^+^ solution already added with DND, the Cs^+^ removal efficiency was only 55%. This fact suggests that formation of Cu-PB in the presence of Cs^+^-adsorbed DND is essential to high removal efficiency. From the optimized addition order of DND, CuCl_2_ and K_4_[Fe(CN)_6_] to a Cs^+^ solution, it seems that the reaction first starts with adsorption of Cs^+^ and then Cu^2+^ to DND, which then reacts with potassium ferrocyanide added to the solution, to form Cs^+^-incorporated Cu-PB@DND precipitate. Such a reaction mechanism suggests that adsorbed Cs^+^ and Cu^2+^ ions react with potassium ferrocyanide in solution, as if the cations were free ions in solution. The adsorbed metal ions and metal complexes obviously enhance aggregation and precipitation of DND, possibly by electrostatic attraction and bridging of several DND particles. The progress of the reaction is shown in Fig. 6.

The present experiment showed that DND did not adsorb appreciably either preformed [Cu_x_Fe_1−x_(CN)_6_]^4−^ or Cs_4_[Cu_x_Fe_1−x_(CN)_6_] in the solution, since Cs^+^ removal efficiency was obviously decreased when DND was added after CuCl_2_ and potassium ferrocyanide. Copper ion must be added after DND and prior to potassium ferrocyanide. Formation of red-brown [Cu_x_Fe_1−x_(CN)_6_]^4−^ was hindered, when potassium ferrocyanide was added after DND and prior to copper chloride. It seems that ferrocyanide ion is adsorbed on Cs^+^-adsorbed DND but does not react with copper ion, in the adsorbed state.

The final precipitation efficiency is obviously promoted by the presence of DND. The final supernatant after overnight standing was colorless and transparent, and the precipitate was brown.

### Removal of Cs^+^ in diluted artificial seawater by using Cu-PB@DND in batch mode

Removal efficiency of Cu-PB@DND adsorbent in batch mode was examined by using 0.015 g of the adsorbent added to 80 mL of Cs^+^ solution in artificial seawater (0.07%). The solution was stirred for 6 hours. Clear supernatant was obtained after the solution was left standing overnight. The initial Cs^+^ concentration was varied in the range of 1 to 150 ppm, and Cs^+^ concentration of the supernatant was measured with ICP-MS. The results are shown in Table 2 for several different initial Cs^+^ concentrations.

**Table 2:** Distribution coefficient (K_d_) and removal efficiency (R_eff_) of Cs^+^ in co-precipitation and batch modes.

mode and adsorbent	Cs^±^ concentration (ppm)		K_d_ (mL/g)	R_eff_ (%)
	initial C_0_	supernatant C_e_		
**co-precipitation mode**				
DND + Cu-PB	6.6	0.003	8.8 × 10^7^	>99
DND only	6.6	4.1	2.4 × 10^4^	38
Cu-PB only	6.6	1.5	1.4 × 10^5^	77
**Batch mode**				
Cu-PB@DND	1	0.004	1.3 × 10^6^	>99
	10	0.041	1.3 × 10^6^	>99
	50	0.22	1.2 × 106	>99

### Calculation of Distribution Coefficient (K_d_), Removal Efficiency (R_eff_), Adsorption Capacity (Q_e_), and Adsorption Isotherm

The data of Cs^+^ removal experiment were analyzed following the equations below. The distribution coefficient K_d_ (mL/g) was calculated according to equation (1).

1 $${{\rm{K}}}_{{\rm{d}}}=({{\rm{C}}}_{{\rm{0}}}\mbox{--}{{\rm{C}}}_{{\rm{e}}}){\rm{V}}/({{\rm{C}}}_{{\rm{e}}}{\rm{M}})$$

where C_0_ and C_e_ are initial and equilibrium concentrations of Cs^+^, and V and M are volume of solution (mL) and weight of adsorbent (g), respectively.

Removal efficiency R_eff_ (%) was calculated according to equation (2).

2 $${{\rm{R}}}_{{\rm{eff}}}=100\times ({{\rm{C}}}_{0}\mbox{--}{{\rm{C}}}_{{\rm{e}}})/{{\rm{C}}}_{0}$$

Equilibrium adsorption capacity Q_e_ (mg/g) is defined as equation (3).

3 $${{\rm{Q}}}_{{\rm{e}}}=({{\rm{C}}}_{0}\mbox{--}{{\rm{C}}}_{{\rm{e}}}){\rm{V}}/{\rm{M}}$$

Although most K_d_ values are reported for a batch mode in literatures, K_d_ value of the present co-precipitation method was calculated by using the data of run 3 in Table 1 and equation (1), assuming 2 mg of adsorbent (calculated from the concentrations of DND, CuCl_2_ and potassium ferrocyanide and the solution volume of 80 mL used in the experiment). The K_d_ value obtained was 8.8 × 10^7^ mL/g (Table 2). Considering that reported K_d_ values for PB and PB analogue adsorbents in batch mode are in the range of 10^3^ to 10^6^ mL/g, depending on the hetero metal M in [M_x_Fe_1−x_(CN)_6_]^y−^ and on the support bead material (silica, alumina, polymers etc.)^13,19,20,25–28,31,38,43–45^, the K_d_ value of the present co-precipitation method is remarkably high, which is due to precipitation and aggregation effect of DND in co-precipitation mode. Co-precipitation effect gives often better removal efficiency than batch mode. When only DND is added to a Cs^+^ solution in Table 1, K_d_ is 2.4 × 10^4^ mL/g, which is still a significantly high value. The K_d_ and R_eff_ values of both co-precipitation and batch modes are summarized in Table 2.

**Figure 7:**
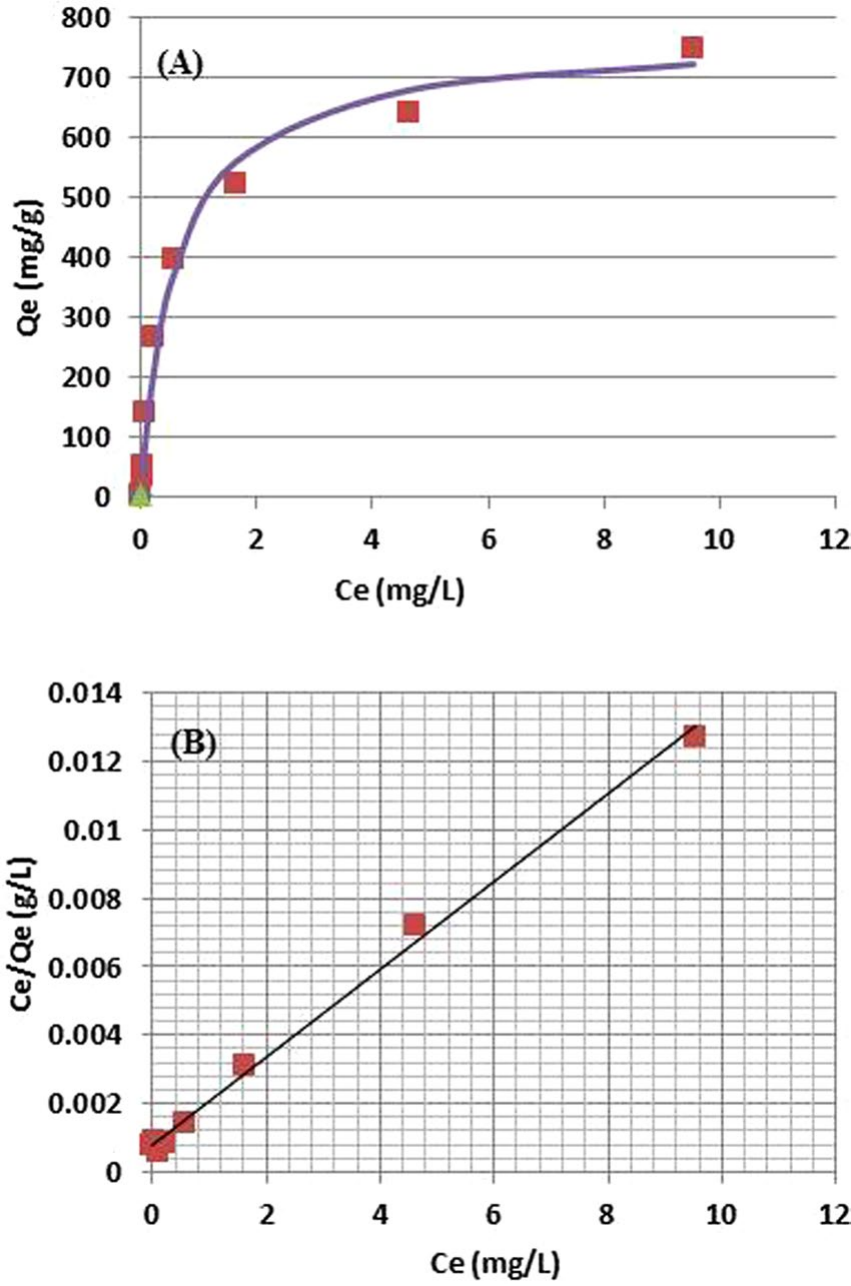
Langmuir plot of the Cs^+^ adsorption data for Cu-PB@DND. The data points are shown with red rectangles, and the calculated best fit curve (**A**) and linear line (**B**) are shown. (**A**) and (**B**) are calculated for an identical data set.

For evaluation of adsorption capacity and for understanding of the adsorption mechanism of Cu-ferrocyanide@DND in batch mode, adsorption data were analyzed based on Langmuir adsorption model, and a good fit was found. From the fitting calculation, maximum adsorption capacity (Q_max_) of the adsorbent was obtained. This value provides estimate of the adsorbent amount required to remove a unit mass of pollutant (Cs^+^) under the system conditions. The data and the calculated curves of C_e_ vs. Q_e_ and C_e_ vs. C_e_/Q_e_ are shown in Fig. 7(A and B), respectively, in which Q_e_ is equilibrium adsorption capacity (mg/g), i.e., Cs^+^ concentration in adsorbent at equilibrium (Cs^+^ in mg/adsorbent in g). In Fig. 7(A), Q_e_ increases rapidly with increasing C_e_ up to around 5 ppm, even after this point Q_e_ increases but much gradually.

Langmuir adsorption equation is expressed in equation (4), which is derived from a model that adsorption takes place on surface monolayer.

4 $${{\rm{Q}}}_{{\rm{e}}}={{\rm{Q}}}_{{\rm{\max }}}\frac{{{\rm{K}}}_{{\rm{L}}}{{\rm{C}}}_{{\rm{e}}}}{1+{{\rm{K}}}_{{\rm{L}}}{{\rm{C}}}_{{\rm{e}}}}$$

In equation (4), K_L_ (L/mg) is Langmuir constant related to energy of adsorption and desorption, and Q_max_ is maximum adsorption capacity (mg/g). Equation (4) can be transformed to equation (5), which indicates that K_L_ and Q_max_ can be calculated from the intercept and slope of Fig. 7(B).

5 $${\rm{Ce}}/{\rm{Qe}}={\rm{1}}/({{\rm{K}}}_{{\rm{L}}}{{\rm{Q}}}_{{\rm{\max }}})+({\rm{1}}/{{\rm{Q}}}_{{\rm{\max }}}){\rm{Ce}}$$

The calculated K_L_ and Q_max_ of Cu-PB@DND are listed together with the fitting residual R^2^ in Table 3. Maximum adsorption capacity Q_max_ and K_d_ of other adsorbents in literature are also shown in Table 3 for comparison. Obviously Q_max_ of the present adsorbent is remarkably high compared to those of simple PB, PB analogues and other common adsorbents. Recently nanocarbon materials such as CNT, graphene, graphene-oxide and reduced graphene-oxide are reported as solid support for PB and PB analogues in Cs^+^ removal^38,47,49^. The performance of these new adsorbents are also included in Table 3. The new adsorbents have notably high Cs^+^ adsorption capability. Another very new adsorbent CNF/PB/PVA, prepared from cellulose nanofiber (CNF) as a PB support with polyvinylalcohol (PVA) as a binder, gives high performance for Cs^+^ adsorption both in batch and column mode^44^.

**Table 3:** Experimental and Langmuir isotherm parameters of Cu-PB@DND for Cs^+^ adsorption and those of related adsorbents in literatures. The values not shown in the table are not reported.

adsorbent	KL (L/mg)	Qmax or Qe (mg/g)	R^2^	K_d_ (mL/g)	reference
Cu-PB@DND in 0.07% artificial seawater	1.63	759	0.996	1.3 × 10^6^	present work
PB/reduced graphene oxide in water	0.017	18.67		4	^37^
PB/CNT in water				4.5 × 10^8^	^38^
PB-Cu-EDTA/silica in 0.45% Na^+^		21.7		2.4 × 10^5^	^31^
Cu-PB polymer/silica in water		20.0		6.7 × 10^6^	^27^
Macrocycle/silica in water	1.79	86.28			^33^
Cu-PB nanoparticle in water				1.6 × 10^6^	^45^
PB/MNC^a^ in 0.3% Na^+^	1.0955	45.87		33471	^43^
CNF/PB/PVA^b^ in water		139		2 × 10^5^	^44^
Hollow PB in water		262.0			^13^
KNiFC^c^ in water pH5.5		520		2.8	^20^
NiFC co-precipitation in alkali water				2 × 10^6^	^17^
AMP-PAN^d^ in seawater		256.0			^46^
Zeolite A in water		222.9			^47^

^a^MNC; magnetic nanoclusters. ^b^CNF; cellulose nanofiber.

^c^KNiFC; potassium nickelferrocyanide. ^d^AMP-PAN; ammonium molybdophophate.

### Mechanism of Cs^+^ adsorption on Cu-PB@DND

Several mechanisms of Cs^+^ adsorption on PB and Cu-PB are reported, including Cs^+^ substitution into the lattice void and Cs^+^ entrapment during complex lattice formation, and the kinetics is not straight forward. The mechanism of Cs^+^ adsorption on Cu-PB@DND is much more complicated and not clear. The elemental composition (wt%) of Cu-PB@DND is C 54.9, Cu 21.7, Fe 13.2, N 8.3, O 1.9, and K 0.2. The Cu and Fe values are significantly higher than previous metal ion adsorption values, for instance, Cu^2+^ 1.78 and Fe^2+^ 1.15 wt% on DND^27^. In other literatures, maximum Cu^2+^ adsorption on DND is reported to be 7.5 wt%^48^ and 5μmol/g^49^. The latter value was obtained in the existence of SO_4_^2−^ for DND from the same source, ALIT, Ukraine, for air-sintered sample similarly to DND(II).

With the specific surface area of 191 m^2^/g and number of acidic groups per area, 0.56 group/nm^2^ reported in the literature for DND of ALIT^49^, the maximum Cu^2+^ adsorption on DND can be calculated to 1.13 wt%, by assuming that all the acidic groups are occupied with Cu^2+^. This value is appreciably lower than the analytical Cu^2+^ value of 21.7 wt% for Cu-PB@DND. From this fact we have an image of Cu-PB@DND that substantially thick Cu-PB layer covers multiple DND particles, and the adsorbent particle is actually something like a mixture of Cu-PB and DND rather than aggregated DND particles with thin surface layer of Cu-PB.

In the mechanistic Cs^+^ adsorption study on Cu_2_[Fe(CN)_6_], the maximum atomic ratio of Cs/Fe is reported to 1.5^30^. Assuming that all the Cu-PB in Cu-PB@DND participate in Cs^+^ adsorption with the ratio of 1.5, the maximum Cs^+^ adsorption on Cu-PB@DND was calculated to 314 mg/g.

On the other hand, maximum ion-exchange capacity of 2.0 mmol Cs^+^/g is reported for DND of ALIT^49^, which corresponds to 127 mg/g Cs^+^ adsorption per Cu-PB@DND, assuming 47.8 wt% DND carbon in the adsorbent (DND C amount was estimated by subtracting cyanide C amount corresponding to N amount, from the total C amount). Addition of the two values of 314 and 127 mg/g amounts to 441 mg/g. This kind of calculation might be somewhat speculative, but explains the high Q_max_ (mg/g) of 759 mg/g in Table 3. The remaining difference between 441 and 759 might be caused by errors in the cited literature values, experimental error of the elemental analysis, or existence of unexplained new mechanism specific to DND, such as formation of Cu-PB together with trapping Cs^+^ in the DND hydrogel within the adsorbent. Hydrogel of DND would facilitate Cs^+^ ion transfer deeper into the inner site of the adsorbent, thus increase the capacity. In silica gel, Cs^+^ adsorption on PB is reported to proceed in a very different manner from usual PB adsorption, and yet Langmuir fitting is observed^27^.

### Removal of Cs^+^ in soil-treated wastewater

Wastewater (A) containing 5 w/v % fine clay powder was obtained after washing contaminated soil sample with high-temperature and high-pressure water. Then the wastewater (A) was treated with the present co-precipitation method, i.e., DND(II), CuCl_2_, and potassium ferrocyanide were added in this order to wastewater (A) with stirring. The wastewater was left standing for settling of the precipitate. In some cases, the supernatant was filtered to give a dark-brown transparent filtrate, which was subjected to ^137^Cs γ-ray measurement. The results are shown in Table 4.

**Table 4:** Removal of Cs^+^ in soil-treated wastewater (A) under various addition conditions of DND, CuCl_2_ and, K_4_[Fe(CN)_6_], and use of coagulant and filtration.

run#	DND (mg/L)	CuCl_2_ (mM)	K_4_[Fe(CN)_6_] (mM)	coagulant	filter *ϕ*(0.22 μm)	Cs^137^ (Bq/kg)
1	0	0	0	−	−	1075
2	0	0	0	+	+	87
3	2.5	0	0	+	−	138
4	2.5	0.5	0.25	+	+	N.D.^a^
5	2.5	2.5	1.25	+	+	N.D.

^a^Not detected.

As shown in Table 4, most Cs^+^ was removed by addition of only DND(II), CuCl_2_, and potassium ferrocyanide. When coagulant and a filter were applied in addition, Cs^+^ level was completely decreased to N.D. (runs 4 and 5).

Another wastewater sample (B) contained fine soil at 3.2 wt/v %. Wastewater (B) was a turbid dispersion, and the soil powder never precipitated even after 12 hours standing. The sample was subjected directly to Cs^+^ co-precipitation procedure. After the procedure, the supernatant was still turbid in most cases. The Cs^+^ concentrations of the supernatants were measured with a ^137^Cs γ-ray spectrometer. The results are shown in Table 5.

**Table 5:** Results of Cs^+^ removal in soil-treated wastewater (B) under various conditions of reagents, coagulant and a filter, showing the effect of DND for rapid soil precipitation.

run #	DND	CuCl_2_	KFC^a^	coagulant	precipitation time	filter	Cs activity (Bq/L)	
	(mg/L)	(mM)	(mM)	(mg/L)	(min)	(1 μm)	Cs 134	Cs 137
1	0.0	0.0	0.0	0.0	>720	−	116	84.6
2	0.0	0.0	0.0	0.0	>720	+	N.D.^b^	N.D.
3	0.0	0.0	0.0	2.5	>720	−	─^c^	─
4	2.5	0.0	0.0	0.0	>720	−	─	─
5	2.5	0.0	0.0	2.5	>720	−	─	─
6	2.5	0.5	0.25	2.5	30	−	7.1	5.5
7	2.5	0.5	0.25	2.5	30	+	N.D.	N.D.
8	0.0	0.5	0.25	0.0	30	+	N.D.	N.D.
9.	2.5	0.5	0.25	0.0	5	+	N.D.	N.D.
10	0.0	0.5	0.25	2.5	10	+	N.D.	N.D.

^a^Potassium ferrocyanide,

^b^Not detected.

^c^Not measured, since precipitation was incomplete at 720 minutes.

**Figure 8:**
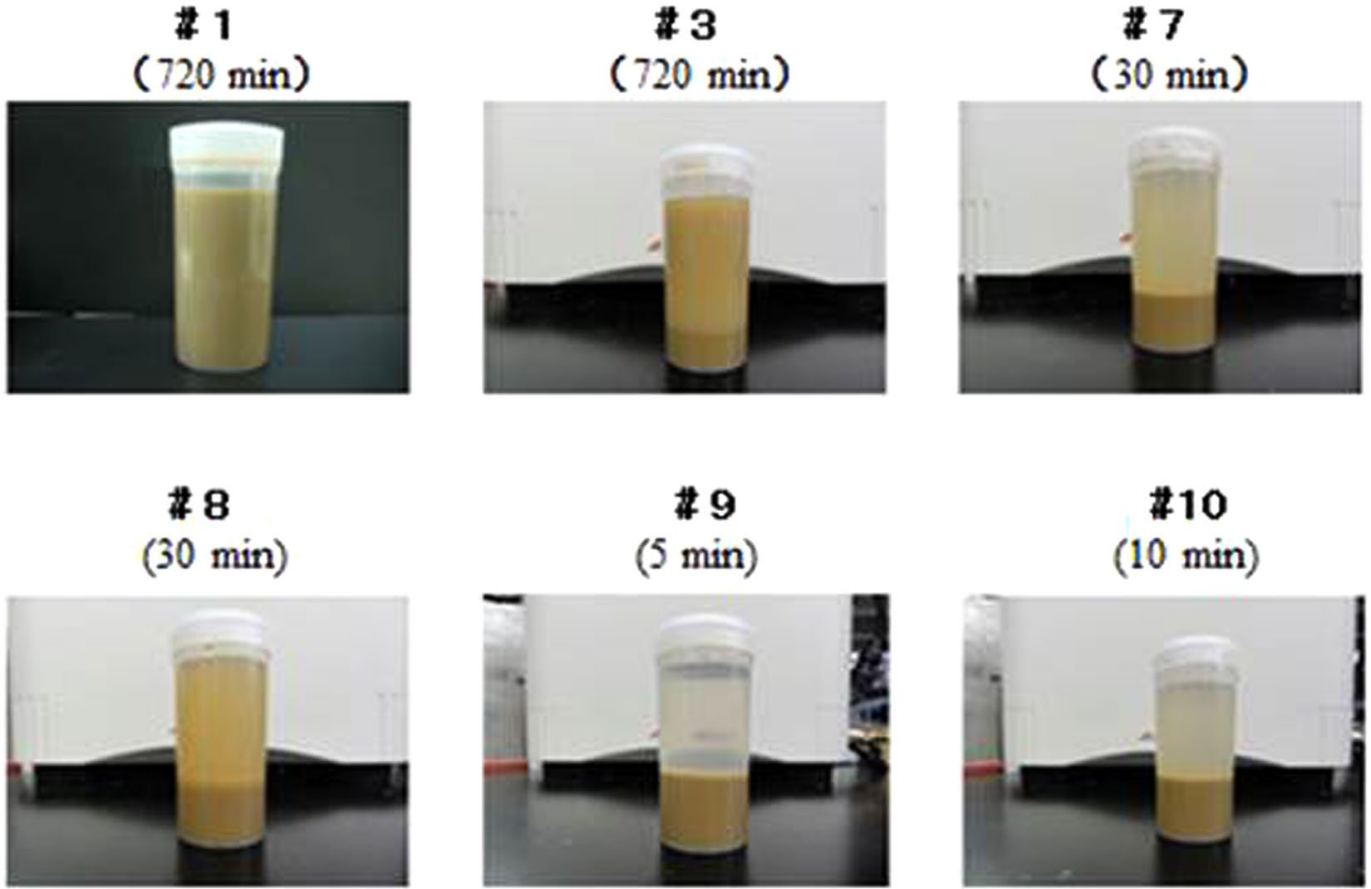
Photos of wastewater (**B**) after treatment with DND, CuCl_2_ and potassium ferrocyanide under various conditions. The time in parenthesis is elapse time after the reagents were added. Numbers of the solutions correspond to those in Table 5.

The effect of precipitation time of soil powder by using Cu-PB@DND was measured. In Fig. 8 are shown the photos of the soil solutions, which show how precipitation became rapid by addition of DND, CuCl_2_ and potassium ferrocyanide.

In Fig. 8, sample # 9 almost completely precipitated only in 5 minutes, and the transparent supernatant was obtained, whose Cs^+^ concentration was below detection limit (Table 5). Other solutions without DND addition in Fig. 8 are very turbid even after 720 minutes standing.

The concentrations of Sr^+^, Ca^2+^, Cu^2+^, Fe^3+^, K^+^, Mg^2+^, Na^+^, S and Si, in the supernatants were measured with ICP-AES, and are listed in Table S2. The table shows that Cu^2+^ and Fe^2+^ were not detected under any condition, whereas Ca^2+^, Mg^2+^, Na^+^ and K^+^ increased significantly by the treatment, which would be due to leaching from the soil powder.

## Conclusions

Detonation nanodiamond (DND) is highly effective for Cs^+^ removal when used together with Cu-PB. The co-precipitation method and the adsrobent Cu-PB@DND do not lose adsortion capability even in diluted seawater and fine soil-containing wastewater. The adsorbent Cu-PB@DND has a distinctly high K_d_ of 1.3 × 10^6^ mL/g and capacity Q_max_ of 7.59 × 10^2^ mg/g. Several other remarkable points of the present method are: (i) excess Cu^2+^ and Fe^2/3+^ added to the solution are not detected in the supernatant. (ii) the efficiency of Cu-PB@DND has no pH dependence, (iii) Cu-PB@DND retains its high removal efficiency even in diluted artificial seawater and soil-containing wastewater. The high capacity is explained with high specific surface area and high adsorption capacity of DND, together with well-known capacity of Cu-PB.

## Experimental

### Surface treatment of DND

Two differently surface-treated DNDs were prepared. In one treatment, DND was sintered in air at 400 °C for 8 hours, and in the other, DND was treated with strongly alkali solution as follows; 36 g of 10 wt% DND aqueous suspension and 24 g of 25 wt% NaOH aqueous solution were mixed and heated at 100 °C for 24 hours. The pH of the suspension was then adjusted to 7 by repeated addition of water, stirring, and decantation of the supernatant. The precipitate was dried by heating at 80 °C for 10 hours and then 200 °C for 1 hour.

The powder DNDs thus prepared were dispersed to water by vigorous sonication to make 1 wt% dispersions. For comparison of Cs^+^ removal efficiency, 1 wt% dispersions of DND(I), DND (II) and DND (III) were used in co-precipitation mode.

### Reagents and samples

Water dispersion of DND(I) (1 wt%) from Vision Development Co., Ltd. Japan, was used as received. The dispersion was sonicated vigorously for a few minutes before use. Diluted artificial seawater was prepared by dissolving commercial natural seawater salt powder (“Nuchimasu” from Nuchimasu Co., Japan) into pure deionized water to 0.07 wt/v%. This concentration was the maximum concentration, at which Cs^+^ could be measured on ICP-AES without Na^+^ interference. To the diluted seawater, commercial standard 1000 ppm non-radioactive Cs^+^ solution (Wako Pure Chemical Industries Ltd., Japan) was added to desired concentrations (pH 7.6). For measurement of Cs^+^, both ICP-AES and ICP-MS were used.

Two soil-treated wastewaters (A) and (B) were obtained from two different spots near Fukushima prefecture. Sample (A) was a suspension of 5 wt/v% soil. Wastewater (B) was a supernatant of wet agricultural soil and had 3.2 wt/v% fine soil powder.

### Instruments

The ICP-AES was SPS5510 from SII Co., Japan with a nebulizer for dispersion solution. The ICP-MS was 7700 × from Agilent. The ^137^Cs γ-ray activity was measured on a Ge semiconductor detector from Ortec Co. The FT-IR spectra of DND were measured as KBr pellet on JASCO FT/IR-6100 at 120 °C under N_2_ to avoid humidity for non-treated DND(I) and at ambient temperature in air for surface treated DND(II) and DND(III). Zeta-potential of DND was measured in 0.1 wt% aqueous solution with DLS (dynamic light scattering) on Zetasizer NanoZS from Malvern Instruments Ltd. The particle size distribution was measured with DLS on a UPA-EX from NIKKISO, Japan. Scanning electron microscopy (SEM) with elemental analysis (EDS) was JSM-7001F from JEOL and INCA x-act from Oxford Instruments.

### Preparation of Cu-PB@DND adsorbent

To 40 mL of water was added 0.4 mL of ultrasonicated 1% DND(II) dispersion and the solution was stirred for a minute. To the solution was added 0.4 mL of 0.1 mol/L CuCl_2_ solution, and the solution was stirred for 5 minutes. After stirring, 0.4 mL of 0.05 M of K_4_[Fe(CN)_6_] was added and the solution was stirred for 1 hour. The solution was left standing overnight for precipitation. The supernatant was removed by pipetting, and the wet precipitate was used for Cs^+^ adsorption experiment. For SEM and IR measurements, the adsorbent was dried at room temperature.

### Procedure for cesium removal in diluted artificial seawater

Cesium removal in seawater was carried out in two modes; a co-precipitation mode using either one of DND(I), DND(II) or DND(III), and the other is a batch mode using Cu-PB@DND adsorbent.

The procedure of co-precipitation was as follows. To 80 mL of 0.07 wt/v % sea water containing 6.6 ppm Cs^+^, was added 2 mL of 1% DND(II) dispersion and the solution was stirred for a minute. The solution became gray and turbid. To the solution was added 0.2 mL of 0.02 mol/L CuCl_2_ solution, and the solution was stirred for 5 minutes. After stirring, 0.2 mL of 0.01 mol/L of potassium ferrocyanide was added and the solution was stirred for 30 minutes. The solution was left standing for 30 minutes, to settle the red-brown precipitate. Colorless supernatant was taken and measured for Cs^+^, Cu^2+^, Fe^3+^, Na^+^ and other metal ions with ICP-AES or ICP-MS.

For comparison, either CoCl_3_ · 6H_2_O, FeCl_3_, or ZnCl_2_ was added instead of CuCl_2_ at the same molar concentration, and the above Cs^+^ removal treatment was performed. Since CuCl_2_ gave the best removal efficiency, all of the following experiments were carried out with CuCl_2_. The total weight of the air-dried final precipitate using CuCl_2_ was 16 mg.

In the batch mode, 15 mg (dry base) of Cu-PB@DND adsorbent was added to 80 mL of 0.07% seawater containing 1 to 150 ppm of Cs^+^, and the solution was stirred for 6 hours. The solution was left standing overnight and the supernatant was measured for Cs^+^ on ICP-MS.

### Procedure for cesium removal in soil-treated wastewaters (A) and (B)

The co-precipitation procedure was performed to wastewaters (A) and (B) under several different conditions. In wastewater (A), either a coagulant (HimolocSS100 from HYMO CORPORATION) at 500 ppm, or a filter of ***ϕ*** 0.22 μm, or both were used to remove fine soil particles in the supernatants. The ^137^Cs γ-ray activity of the supernatants was measured for both samples, in addition, ^134^Cs was also measured for sample (B). For wastewater (B), a filter of ***ϕ***1 μm was used.

## Electronic supplementary material

Supplementary Information
