# Supplementary material for: A Highly Efficient Adsorbent Cu-Perusian Blue@Nanodiamond for Cesium in Diluted Artificial Seawater and Soil-Treated Wastewater
Source: Sci Rep. 2018 Apr 11;8:5807. doi: 10.1038/s41598-018-24129-0 (PMC5895765; doi:10.1038/s41598-018-24129-0)
Supplement: Supplementary file 1 — Supplementary Information [file 41598_2018_24129_MOESM1_ESM.docx]

Supplementary Information to:

A Highly Efficient Adsorbent Cu-Perusian Blue@Nanodiamond for Cesium in Diluted Artificial Seawater and Soil-Treated Wastewater

Kazuko Matsumoto^1,*^, Hideyuki Yamato^2^, Seishiro Kakimoto^2^, Takeshi Yamashita^3^, Ryutaro Wada^4^, Yoshiaki Tanaka^5^ , Masakazu Akita^2^ & Tadamasa Fujimura^2^

^1^Department of Applied Chemistry, Tokyo University of Technology, 1404-1, Katakura-cho, Hachioji, Tokyo 192-0982, Japan. ^2^Vision Development Co. Ltd., 2-8-21, Kikuya bld., Kyobashi, Chuo-ku, Tokyo 104-0031, Japan. ^3^Mechanical Engineering Research Laboratory, Kobe Steel, Ltd., 1-5-5, Takatsukadai, Nishi-ku, Kobe 651-2271, Japan. ^4^Natural Resources & Engineering Business, Kobe Steel, Ltd., 9-12, Kita-Shinagawa, 5-Chome, Shinagawa-ku, Tokyo 141-8688, Japan. ^5^Nuclear & CWD Division, Natural Resources & Engineering Business, 2-7, Iwaya-Nakamachi, Nada-ku, Kobe 657-0845, Japan. *Correspondence should be addressed to K. M. ([kmatsu@yf6.so-net.ne.jp](mailto:kmatsu@yf6.so-net.ne.jp))

run

conc. in supernatant (ppm)

**#**^a^ Na K Mg Ca

1 175 5.86 13.6 3.49

2 177 5.82 13.4 3.47

3 176 5.93 13.3 3.42

4 178 6.02 13.4 3.45

^a^ run numbers correspond to those in Table 1.

**Table S1. Concentration of several metal ions in the supernatant after**

**Cs^+^ removal treatment in 0.07 % seawater with co-precipitation mode.**

run elements in the supernatant

# Sr Ca Cu Fe K Mg Na S Si

1 N.D.^a^ 2.00 N.D. 4.16 1.45 0.90 6.2 4.26 24.41

6 N.D. 11.84 N.D. N.D. 20.76 3.94 8.9 4.48 11.50

8 N.D. 10.39 N.D. N.D. 15.21 3.35 8.0 4.17 13.79

9 N.D. 15.01 N.D. N.D. 20.12 4.94 11.5 4.40 11.18

10 N.D. 12.82 N.D. N.D. 16.95 4.14 13.4 4.36 12.30

a not detected

**Table S2. Concentration (ppm) of several elements in the supernatants of soil-treated wastewater (B) after Cs^+^ removal treatment. The run numbers correspond to those in Table 5.**

**（Ａ）**

**（Ｂ）**

**（％）**

**Particle diameter ((μm)**

**Particle diameter ((μm)**

**（％）**

Figure S1. Particle diameter distribution of (A) DND(II) and (B) Cu-PB@DND.
